# Activity Budget and Behavioral Patterns of Himalayan Musk Deer in Gaurishankar Conservation Area, Nepal

**DOI:** 10.1002/ece3.72766

**Published:** 2026-01-05

**Authors:** Bijay Bashyal, Narayan Prasad Koju, Lila Paudel, Paul Buzzard, Randall C. Kyes

**Affiliations:** ^1^ Central Department of Environmental Science Tribhuvan University Kathmandu Nepal; ^2^ Center for Postgraduate Studies, Nepal Engineering College, Pokhara University Lalitpur Nepal; ^3^ Key Laboratory of Genetic Evolution and Animal Models, Kunming Institute of Zoology, Chinese Academy of Sciences Kunming Yunnan China; ^4^ Department of Psychology University of Washington Seattle WA USA; ^5^ Washtenaw County Conservation District Ann Arbor Michigan USA; ^6^ Departments of Global Health and Anthropology, Center for Global Field Study, Washington National Primate Research Center University of Washington Seattle WA USA

**Keywords:** activity peak, behavioral patterns, camera trapping, conservation, monitoring, seasonal variation

## Abstract

The Himalayan Musk Deer (HMD, *Moschus leucogaster* ) is an endangered species threatened by habitat loss, illegal hunting, and human activities. However, despite its conservation importance, the activity budgets and behavioral patterns of wild populations remain poorly understood, thus representing a key research gap. Using 25 months of camera trap data (October 2018–March 2023), this study investigated the activity patterns, behavior, and temporal overlap of HMD in the Lapchi Valley of Nepal's Gaurishankar Conservation Area. Analyzing 624 images and 323 video clips, we developed an ethogram and estimated behavioral activity budgets across three reproductive periods: pre‐rut, rut, and post‐rut. Behaviors were then categorized and analyzed with BORIS Software and statistical tests, including Chi‐square and Kruskal‐Wallis ANOVA. HMD were predominantly crepuscular, with peaks in activity at dawn and dusk, including a minor nocturnal peak. The range of activity included locomotion (30.68%), standing (28.68%), vigilance (10.72%), sniffing (9.98%), and feeding (9.45%). Changes in behavior were significant across reproductive period, such as increased locomotion during the post‐rut period and increased sniffing bout length in the pre‐rut but not between sexes. Females exhibited significantly higher vigilance, whereas males showed more locomotion during rut, which may be linked to territorial and mating strategies. Increased vigilance during the post‐rut period for both sexes, suggesting a response to livestock presence, and behavioral adjustment to anthropogenic disturbance. The study also documented rare behaviors such as tail‐pasting, courtship displays, acoustic communication, lactation, and alarm responses. Overall, this study provides new insights into the behavioral ecology of Himalayan musk deer in wild and highlights the importance of continuous monitoring. The findings offer valuable guidance for conservation management and the development of policies aimed at minimizing anthropogenic disturbances in musk deer habitats.

## Graphical abstract

The Himalayan Musk Deer (HMD, Moschus leucogaster) is an endangered species that faces threats from habitat loss, illegal hunting, and human activities. Using 25 months of camera trap data (October 2018–March 2023), this study investigated the activity patterns and behavior of HMD in the Lapchi Valley of Nepal's Gaurishankar Conservation Area.

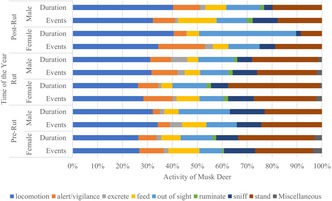

## 1 Introduction

Nepal is home to four species of musk deer, namely, *Moschus leucogaster* , *M. chrysogaster* , *M. fuscus* , and *M. cupreus* (Jnawali et al. 2011; Amin et al. 2018; Singh et al. 2020). Among these, the Himalayan Musk Deer (HMD, *M. leucogaster* ) is distributed widely, but discontinuously, across the central and eastern landscapes of the Nepal Himalayas, inhabiting alpine forests and scrub vegetation between 2200 and 4300 m above sea level (masl) (Green 1986; Mainali et al. 2023; Dhami et al. 2024). All these elusive species face significant survival threats, primarily poaching for their highly valued musk pod (Yang et al. 2003), habitat degradation, and competition with livestock (Green 1986; Subedi et al. 2012). Consequently, musk deer populations have experienced a dramatic decline across their range (Yang et al. 2003).

All four musk deer species are currently classified as Endangered by the IUCN (Timmins and Duckworth 2015b, 2015a; Wang and Harris 2015; Harris 2016; IUCN 2018) and listed under Appendix I of CITES (CITIES 2023). In Nepal, they are listed as a protected species under the National Parks and Wildlife Conservation Act (1973) and categorized as Endangered (EN) under the National Red List (Jnawali et al. 2011). This precarious conservation status emphasizes the urgent need for conservation intervention. Understanding the behavioral ecology of musk deer is particularly critical, as it informs conservation interventions such as population monitoring, disturbance management, anti‐poaching operations, human‐wildlife conflict mitigation strategies, and spatial planning (Timmins and Duckworth 2015a).

Behavioral studies offer valuable insights into animal ecology, providing crucial information for conservation planning (Sutherland 1998). Activity budgets, which quantify how animals allocate time to different behaviors, are used to evaluate the impact of environmental disturbances, evaluate animal welfare, and understand differences based on sex and other factors (Hamel and Côté 2008; Christiansen et al. 2013; Howell and Cheyne 2019). These studies also provide a baseline for comparison between wild and captive populations, thereby informing both in‐situ and ex‐situ management (Miller et al. 2020). Despite the importance of such research, behavioral studies of musk deer are limited. Current understanding of musk deer behavior largely stems from studies conducted in captivity (Meng et al. 2008; Lu et al. 2009), with only a few studies exploring their behavioral patterns in the wild. For example, Singh et al. (2022) focused exclusively on latrine‐specific behaviors of musk deer in the Annapurna Conservation Area, and Tran et al. (2025) documented mating behavior of musk deer in northern Vietnam.

Behavioral patterns and activity budgets are known to vary with the time of day and/or year and are influenced by various intrinsic and extrinsic factors, including age, sex (Huettner et al. 2021), climatic conditions (Pęksa and Ciach 2018), and food availability (Albani et al. 2020). Additionally, anthropogenic disturbances such as construction projects and tourism have been reported to significantly alter animal behavior in the wild (Jiang et al. 2013; Pęksa and Ciach 2018; Albani et al. 2020). Lapchi valley, part of the Gaurishankar Conservation Area (GCA), Nepal is experiencing increasing anthropogenic pressures in recent years from livestock grazing, road construction, and hydropower development (Ghimire and Phuyal 2022; Tiwari 2022; Koju et al. 2024). Livestock grazing, which is permitted under Nepal's Himalayan National Park Regulations (1979), has intensified habitat overlap between domestic animals and musk deer, while infrastructure expansion poses a direct threat to their habitats. Such factors can substantially alter the animals' behavior. Prior studies in GCA have demonstrated that HMD signs occur more frequently away from human settlements (Koju, Bashyal, and Shah 2021), highlighting their sensitivity to disturbance. Therefore, continuous monitoring of the activity and behavior of endangered species like musk deer is essential to inform adaptive management strategies (Worku et al. 2021).

Musk deer are solitary, territorial, and shy, with a core area of 0.15–0.31 km^2^ (Sathyakumar et al. 1992). They are known to communicate through scent marking and exhibit seasonal migratory patterns (Green 1987). These cryptic, solitary behaviors make field observation difficult. Camera trapping has emerged as an effective tool for monitoring elusive species, enabling researchers to document behavioral activities and develop ethograms (Ghaskadbi et al. 2016; Koju et al. 2024).

The current study utilized camera trap records (images and video) collected over multiple years to examine the behavioral activity budget and daily activity patterns of HMD in the Lapchi Valley of GCA. We constructed a behavioral inventory of wild HMD, estimated activity budgets and daily activity patterns across pre‐rut, rut, and post‐rut reproductive phases, and evaluated the implications of these behavioral patterns for musk deer conservation. This study provides critical baseline information to guide evidence‐based conservation strategies for endangered HMD.

## 2 Materials and Methods

### 2.1 Study Area

**FIGURE 1:**
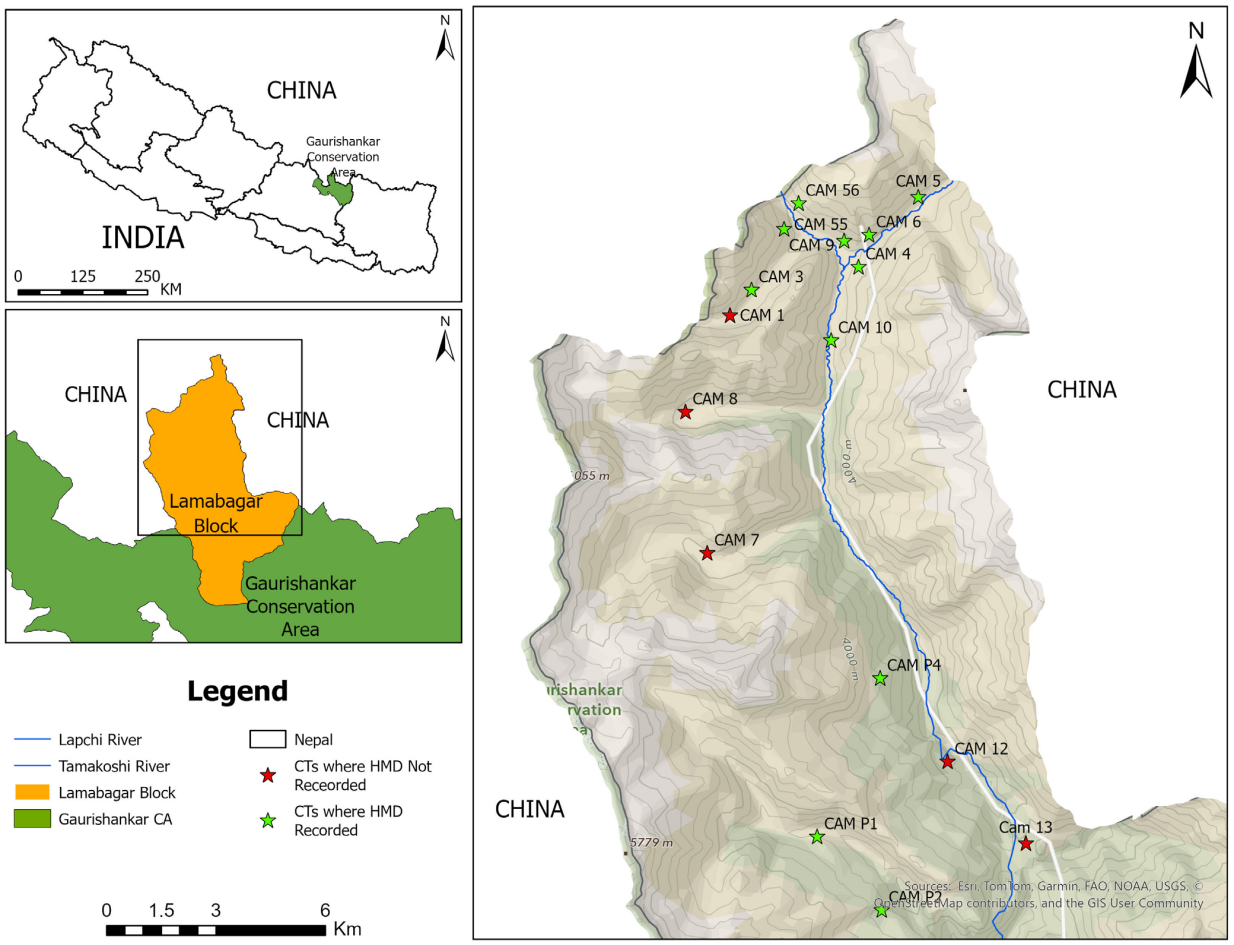
Map of the Lapchi Valley study area showing camera trap locations and those where Himalayan Musk Deer presence was recorded.

The study was conducted in the Lapchi Valley of Dolakha district of Nepal, within the GCA. The GCA is one of the newest protected areas in the country and spans an area of 2179 km^2^ and extends from 85° 45′ 54 “to 86° 34′ 0” East Longitude and 27° 34′ “to 28° 12′ 30” North latitude (Figure 1). It encompasses three districts of Bagmati Province: Dolakha, Sindhupalchowk, and Ramechhap. The region supports rich floral and faunal diversity, including at least 18 vegetation types ranging from riverine forests to alpine scrub. It supports 77 species of mammals, 252 species of birds, 27 species of reptiles, 12 species of amphibians, and 24 species of fish (NTNC 2013; Chetri et al. 2022).

Lapchi lies at the foot of the Lapchi‐Khang mountain range, a significant pilgrimage destination for Tibetan Buddhists, and renowned for the meditation caves of Jetsun‐Milarepa surrounding “ChöraGephel Ling.” The valley, previously inaccessible due to the lack of modern transportation, has recently been connected via a road to Lamabagar, which has increased accessibility but also increased influx of hydropower development projects and tourism activities. The local Sherpa communities, who practice semi‐nomadic and animal husbandry, rely on seasonal migration and grazing livestock in high elevations during summer and return to the village of Numnan (Lumnan) in winter. As noted earlier, Lapchi Valley supports a diversity of wildlife including the HMD (the only musk deer species found in the area) (Shrestha et al. 2018) and a number of other threatened species such as the Himalayan Black Bear ( *Ursus thibetanus* ), Snow Leopard ( *Panthera uncia* ), and Asiatic Golden Cat ( *Catopuma temminckii* ) (Koju et al. 2020; Koju, Bashyal, Pandey, et al. 2021).

## 3 Field Survey and Data Collection

### 3.1 Camera Trapping

A total of 26 Bushnell Trophy Cam (Model #119537C) camera traps were deployed across 16 locations for 25 months in two phases; phase 1: October 2018–May 2019; and phase 2: October 2021–March 2023, resulting in a total trapping effort of 3156 camera‐trap days (one trap day is equal to one camera active for 24 h). Cameras were installed in grids of 2 × 2 km^2^, targeting mammalian surveillance across various habitats, including forests, scrub vegetation, pasture land, rock, and cliff areas (Koju, Bashyal, and Shah 2021; Koju et al. 2023). The selected 16 camera sites represented the full elevational gradient (3200–4250 m asl) and major habitat types (Betula–Juniper forest, alpine scrub, and talus slopes) used by HMD. Environmental attributes, including elevation, slope, and aspect, are summarized in Table 1. The range of these attributes demonstrates that the sampling adequately covered the species' habitat spectrum and minimized habitat‐specific bias. Cameras were placed 30–40 cm above the ground, depending on the terrain, and programmed in hybrid mode to capture three consecutive photographs followed by a 10‐s video per trigger. The placement of camera traps was focused on animal trails with active signs of wildlife to help maximize detection probability. We acknowledge that this may bias the data towards high‐use areas and potentially overrepresent behaviors expressed along trails. HMD were recorded in 11 camera trap locations, while domestic animals (including humans) were recorded from 15 locations (Figure 1, Table 1).

**TABLE 1:** Elevation, slope, aspect, and landcover type of the camera trap locations where Himalayan musk deer behavior was recorded in Lapchi Valley, Gaurishankar Conservation Area, Nepal, November 2018–March 2023.

Camera ID	Elevation (m asl)	Slope (degree)	Aspect	Landcover type	Location
CAM 10	3206	10	South	Betula‐Juniper Forest	Trail
CAM P4	3593	35	East	Betula‐Juniper Forest	Cliff above the human trail
CAM 4	3842	32	West	Forest	Within forest
CAM 9	3843	15	South	Juniper Forest	Along the yak and human trail
CAM 55	3901	20	North	The thin forest of Betula and *Abies*	Trail beside of riverbank near the livestock shed
CAM 6	4045	10	South	Edge of *Betula‐Rhododendron* Forest	Along the yak and human trail
CAM 3	4103	20	North	Alpine talus	Boulders nearby stream in Alpine
CAM 5	4234	30	South	Scrub land	Cliff above the yak pasture
CAM 56	4098	35	South	Alpine talus	Boulder cliff
CAM P1	3403	25	East	*Betula‐Rhododendron* Forest	Samling area
CAM P2	3323	15	East	*Betula‐Rhododendron* Forest	Near Kukur Raja pass

### 3.2 Behavioral Data

Over the course of the study, we recorded a total of 323 video clips and 624 independent images of HMD. These video clips/images represented independent events as defined by recording photo separated by intervals of 30 min or more between successive captures of the musk deer (Carbone et al. 2001). Video footage documenting the activity of HMD was analyzed for behavioral budgets and daily activity patterns. Additionally, photographs were used for further confirmation. Unclear photos and blank video footage were excluded from the analysis. Among the 323 video clips analyzed, 51 (16.24%) were recorded during the pre‐rut period, 214 (64.64%) during the rut, and 38 (12.95%) during the post‐rut period. Furthermore, out of the total 624 camera‐trap events, 53 (8.49%), 375 (60.09%), and 124 (19.87%) events were observed during the pre‐rut, rut, and post‐rut periods, respectively.

### 3.3 Behavioral Assessment

Captured videos were analyzed using BORIS [Behavioral Observation Research Interactive Software] version 8.20.4 (Friard and Gamba 2016). The video data were analyzed by applying focal animal sampling (Altmann 1974; Seyrling et al. 2022) for nine behavioral categories, which were identified based on prior studies (Meng et al. 2008; Meng et al. 2011; Singh et al. 2022). Behaviors were coded, and sex was determined based on visible outgrown canines (male). For individuals with unclear facial markings, we compared consecutive photos (captured in the hybrid setting before the video recording) to identify the sex (Table 2). Individuals with unclear identity were excluded from analysis.

**TABLE 2:** Ethogram describing behavioral categories exhibited by Himalayan musk deer in the Lapchi Valley, Gaurishankar Conservation Area, Nepal.

Behavior	Definition
Feed	Ingesting or chewing leaves, grass, or moss
Excrete	Defecating or urinating
Sniff	Exploring the ground with nose while stationary or walking
Locomotion (walk/run)	Walking or running
Stand	Standing with or without alertness to external stimuli
Alert	Focusing on potential threats like predators, domestic animals, people, or other wildlife
Ruminate	Chewing cud while standing, sitting, or walking
Out of sight	Leaving the camera frame by walking or running
Miscellaneous	Other activities, including tail pasting, scrapping, etc.

The number of behavioral events was recorded, and the percentage of behavioral events was calculated as the number of observations (i.e., events) of a specific type of behavior divided by the total number of all behavioral events. Activity budgets were calculated as the proportion of total observed time allocated to each mutually exclusive behavior (Seyrling et al. 2022). Behaviors were grouped into three reproductive periods: the pre‐rut (August–October), the rut (November–January), and the post‐rut period (February–April) based on prior studies (Meng et al. 2008). To avoid bias, a single researcher analyzed all videos.

### 3.4 Activity Pattern

#### 3.4.1 Daily Activity

Independent events were used to assess daily activity patterns. Daily activity was classified as diurnal, nocturnal, or crepuscular based on sunrise and sunset times obtained from https://www.timeanddate.com/ and adjusted for seasonal variation (Noor et al. 2017). Since sunrise and sunset vary seasonally, time windows were adjusted for each reproductive period to ensure accuracy. Independent images of HMD and domestic animals (including humans) were used to estimate the daily activity overlap.

### 3.5 Statistical Analysis

Behavioral event data were summarized as counts and durations, then standardized as percentages to generate activity budgets. Statistical differences in activity budgets between sexes and reproductive periods were tested using Chi‐square tests and Kruskal‐Wallis test when data violated normality and confirmed by Shapiro–Wilk tests. Because the behavioral dataset included unequal sample sizes and several non‐normal distributions, nonparametric tests (Kruskal–Wallis, Chi‐square) were used for primary comparisons. However, for comparative purposes and to explore potential interaction effects between sex and reproductive period, an additional two‐way ANOVA was applied to the summarized mean durations of behavioral categories. Although this parametric test was not the primary inferential approach, it served as an exploratory check to evaluate the consistency of results across analytical frameworks. A regression analysis was conducted to examine the relationship between behavior, activity budgets (seconds), and influencing factors (reproductive period, sex).

The daily activity patterns were analyzed using density estimation with the “overlap” R package. For estimating overlap, all the cases had a sample size of *n* > 50 and the estimation was conducted using dhat4 (Δ4) coefficient of overlap. This coefficient operates on a scale from 0 to 1, where 0 signifies complete segregation, while 1 indicates total overlap in daily activity patterns (Ridout and Linkie 2009; Meredith and Ridout 2014). Analyses were performed in R version 4.2.2 (R Core Team 2022).

## 4 Results

### 4.1 Behavior of Himalayan Musk Deer

We obtained a total of 624 independent camera‐trap events of HMD recorded from a trapping effort of 3156 camera‐trap days throughout the study period. Out of the total 624 camera‐trap events, 53 (8.49%), 375 (60.09%), and 124 (19.87%) events were observed during the pre‐rut and post‐rut periods, respectively.

We analyzed 66.6 min (1.11 h) of video footage across 323 video clips, documenting 942 behavioral events of HMD (Table 3). The most frequently observed behaviors included locomotion (30.25%) and standing (23.57%), followed by alert/vigilance (10.72%), sniffing (9.98%), feeding (9.45%), excretion (2.12%), and rumination (1.8%). Notably, 10.19% of the recordings involved cases where the HMD moved out of the camera frame and thus no behavioral data was available.

**TABLE 3:** Total time allocation and number of events recorded per behavioral category for the Himalayan Musk Deer in Gaurishankar Conservation Area, Nepal.

Behavioral category	Total time (min)	Percent of time spent on behaviors			Total number of events	Percentage of behavioral events		
		Total time	Female	Male		Total events	Female	Male
Locomotion	20.44	30.68	27.59	33.08	285	30.25	28.95	31.44
Standing	19.11	28.68	33.3	25.1	222	23.57	24.28	22.92
Alert	5.41	8.12	7.71	8.42	101	10.72	12.25	9.33
Sniffing	4.1	6.15	6.56	5.84	94	9.98	10.02	9.94
Feeding	3.87	5.81	6.09	5.59	89	9.45	10.02	8.92
Excretion	2.24	3.36	1.19	5.05	20	2.12	1.56	2.64
Ruminate	1.13	1.69	1.73	1.68	17	1.8	1.56	2.03
Miscellaneous	0.67	1.01	0.99	1.03	18	1.91	2.23	1.62
Out of sight	9.65	14.48	14.83	14.21	96	10.19	9.13	11.16
Total	66.62	100.00			942	100		

However, in terms of time allocation (activity budget), HMD (both male and female combined) spent the largest proportion of time in locomotive movements (30.68%), followed by standing (28.68%) (Table 3). We also observed that the number of feeding events (9.45%) exceeded the proportion of time allocated on feeding (5.81%) (Table 3). Statistical analysis showed no significant difference in either activity budget (*U* = 5913.5, *p* = 0.856) or frequency of events (*U* = 5980, *p* = 0.741) between the female and male musk deer, suggesting that both sexes invested similar overall time in activity.

### 4.2 Activity Budget During Different Reproductive Periods

Of the total 66.6 min of videos analyzed, a total of 63.51 min consisted of pre‐rut (7.23 min, 11.39%), rut (45.67 min, 71.92%), and post‐rut (10.6 min, 16.69%). Likewise, 63.51 min resulted in 884 behavioral events—153 (17.31%), 609 (68.89%), and 122 (13.80%) during the pre‐rut, rut, and post‐rut season, respectively, and thus were considered for further analysis.

**FIGURE 2:**
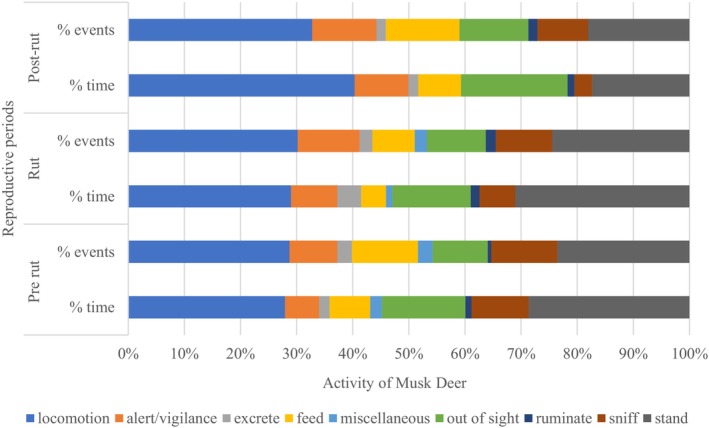
Total number of events (%) and duration of events (%) exhibited by Himalayan musk deer during pre‐rut, rut, and post‐rut periods in Lapchi valley, Gaurishankar Conservation Area, Nepal.

Both frequency and duration of behavioral events varied across the reproductive periods for musk deer (Figure 2). Frequency of behavioral events varied significantly (*N* = 884, *χ* ^2^ = 64.84, df = 2, *p* < 0.001) between sex and reproductive periods (Table S1). Likewise, there were significant differences in the duration of behavioral events between the sex of musk deer and reproductive periods, indicating that male and female musk deer have different behavioral strategies across reproductive periods (Table S2).

However, statistical analysis indicated no significant differences (*N* = 884, *χ* ^2^ = 13.13, df = 16, *p* = 0.66) between behavioral categories and frequency of behavioral events across reproductive periods (Tables S1, S2). There was, however, a significant difference in the duration of time spent (activity budget) on different behavioral categories during different reproductive periods (*N* = 3810.45, *χ* ^2^ = 128.85, df = 16, *p* < 0.001) (Tables S3, S4).

Locomotion was recorded more during the rut and post‐rut periods, with the highest proportion of time spent on this behavior (40.4%) during the post‐rut, likely resulting from territorial and foraging movements.

Post Hoc Tukey tests (following Kruskal–Wallis ANOVA; *N* = 884 Behavioral Events, Df = 2) revealed significant differences in locomotion between reproductive periods (during rut and post‐rut: *M* = −1.497, SE = 0.27, *p* < 0.001, and pre‐rut and rut: *M* = −1.873, SE = 0.27, *p* < 0.001). Similarly, there were significant differences between standing behaviors and periods (during pre‐rut and rut: *M* = −2.017, SE = 0.27, *p* < 0.001 and during rut and post‐rut: *M* = −2.056, SE = 0.27, *p* < 0.001). The duration of sniffing behavior was highest during the pre‐rut period, followed by rut and post‐rut, respectively. However, the duration of alert/vigilance was opposite, with the greatest vigilance observed during post‐rut and the least during pre‐rut (Figure 2). The post‐rut period coincides with the shifting of livestock to musk deer habitats in Lapchi Valley.

### 4.3 Sex Specific and Reproductive Period Behavioral Patterns

**FIGURE 3:**
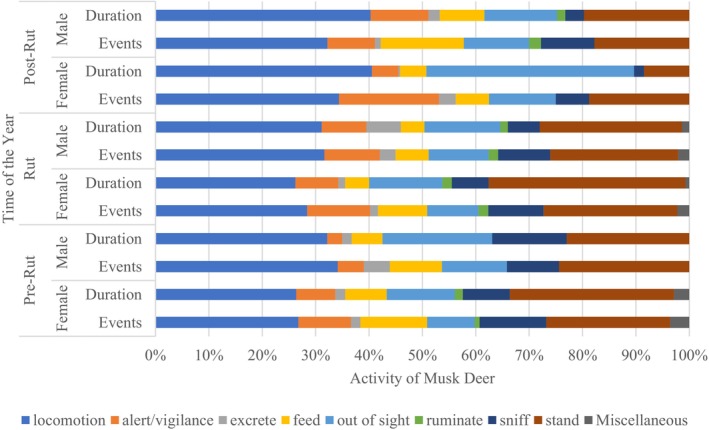
Percentage of total events and duration of behaviors exhibited by female and male Himalayan musk deer during the pre‐rut, rut, and post‐rut periods in the Lapchi Valley of Gaurishankar Conservation Area, Nepal, Oct 2021–March 2023.

Male and female musk deer exhibited distinct behavioral patterns, with females spending more time in vigilance activities than males (Figure 3). The Chi‐square test indicated a significant difference in occurrences of behavioral events during pre‐rut, rut, and post‐rut periods between male and female musk deer (*N* = 884, *χ* ^2^ = 64.83, df = 2, *p* < 0.001). Male musk deer exhibited locomotion more frequently during the rut and post‐rut periods, with the highest proportion of time spent on this behavior (40.4%) during the post‐rut. During pre‐rut and rut periods, females devoted slightly more time to feeding, whereas males exhibited higher feeding activity during the post‐rut period. Sniffing behavior, though similar in percentage of events across periods, showed reduced duration during post‐rut for both sexes. Female musk deer were observed to spend more time on standing during pre‐rut and rut periods, while standing decreased drastically during the post‐rut period. Similarly, males devoted more time to locomotion compared with females during pre‐rut and rut periods. An exploratory two‐way ANOVA based on summarized mean durations of behavioral categories between sex and reproductive period showed no significant interaction between sex and behavioral categories (*F* = 0.229, df = 8, *p* = 0.985) or between sex and reproductive period (*F* = 1.33, df = 2, *p* = 0.268) (Figure 3, Table S6).

### 4.4 Activity Patterns of Himalayan Musk Deer

**FIGURE 4:**
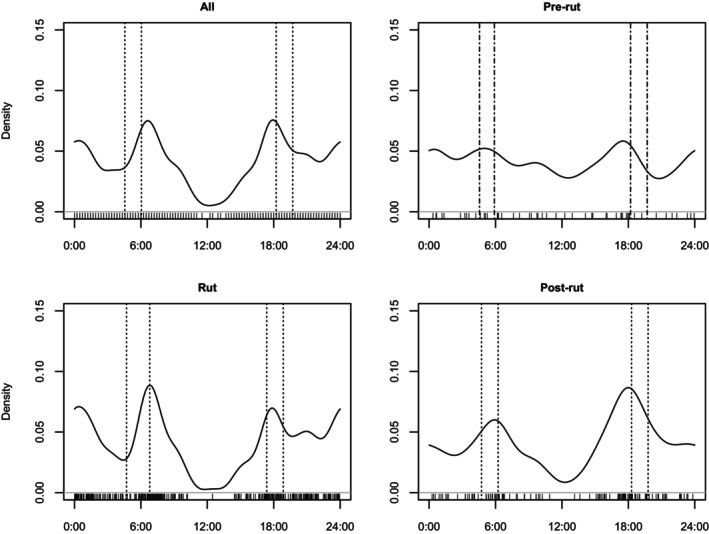
Daily activity pattern of HMD during different reproductive periods in Lapchi Valley, Gaurishankar Conservation Area, Nepal.

During the study, the daily activity of HMD exhibited sharp peaks just after the sunrise and before sunset, resulting in a predominant crepuscular activity pattern. Likewise, the daily activity also showed smaller peaks during midnight, indicating a slight pattern of nocturnal activity as well (Figure 4a).

### 4.5 Activity Pattern Comparisons During Different Reproductive Periods

The daily activity pattern of HMD did not violate the assumption of homogeneity of variance (Levene's test [*p* = 0.188]) and met normality, checked with Q–Q plot. There was no statistically significant difference in the activity of musk deer across the pre‐rut, rut, and post‐rut periods (*F* = 1.386, *p* = 0.251) (Table S5).

During the pre‐rut period, musk deer were observed to engage in activity almost throughout the day (Figure 4b). During the rut period, the daily activity peak was observed during sunrise, while during post‐rut periods the daily activity peak was highest during sunset (Figure 4c,d).

### 4.6 Activity Overlaps Between Musk Deer and Domestic Animals

A total of 422 independent events comprising domestic animals (yaks, horses, dogs) and humans were captured during the study period. A total of 356 events fell within the specific reproductive periods (pre‐rut, rut, and post‐rut) of musk deer under consideration.

**FIGURE 5:**
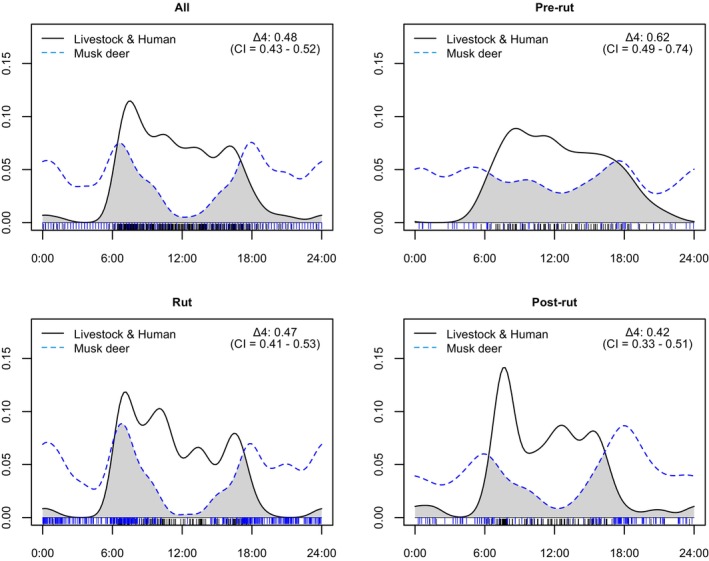
Daily activity overlap between HMD and domestic animals and humans recorded during respective reproductive periods in Lapchi Valley, Gaurishankar Conservation Area, Nepal.

Human and domestic animals were mostly active during the morning hours throughout most of the year, with activity peaking after the sunrise. However, HMD had variable daily activity during each reproductive period (Figure 5).

The highest activity overlap coefficient (∆ = 0.62, CI = 0.49 – 0.74) was observed between HMD and livestock and humans during the pre‐rut period. Likewise, the lowest activity overlap was observed during the post‐rut period (∆ = 0.42. CI = 0.33 – 0.51). During the post‐rut, HMD shifted its daily activity toward sunset, presumably to avoid detection or interaction with domestic animals and humans.

### 4.7 Rare and Miscellaneous Behaviors

Several rarely described behaviors were observed, including self‐grooming (e.g., body shaking), courtship displays, and alarm responses. Unique behaviors such as tail‐pasting (rubbing the tail on vegetation by the male), lip movements, and acoustic signals were also recorded (Table 4).

**TABLE 4:** List of rare and miscellaneous behaviors.

Rare and miscellaneous behaviors recorded	Supporting photo/video ID (selected)
Body shaking (self‐grooming)	Video 11180404, Video 11080350
Courtship displays	Video 11040302
Acoustic signals	Video 11040302
Tail‐pasting	Video 12060698
Alarm behaviors	Video 02030039
Non‐solitary	Video 06220093 Image 06160337; Image 06020195; Image EK000311, Image 08010127
Digging by fore limbs	Video 08180144
Excretion	Video 12010033 (Urinating); Video 12030063 (Defecating)
Nursing	Image 06010177

HMD also exhibited a distinct excretion behavior. They first sniff the ground, likely to find a suitable spot. They then use their forelimbs to scratch or dig the ground. Once the spot is prepared, the deer turns around, bends its hind limbs, and shrinks its body before urinating/defecation. This process suggests a very deliberate and instinctive approach to excretion. Additionally, musk deer were frequently observed shaking their neck/head after sniffing the excrement. However, it was unclear whether this behavior was associated with excretion or was a part of sniffing behavior.

We also documented a novel behavior in HMD referred to as tail pasting, where musk deer were observed rubbing their back against vegetation. During this process, the musk deer slightly raises its back with the help of its hind limbs and shakes its back against the vegetation. Tail‐pasting not only helps mark territory but also serves as a means of olfactory communication.

Notably, HMD displayed alarm behaviors, such as swift running in the opposite direction of their initial movement, potentially triggered by predators or human presence. In one video, a musk deer was observed before running away quickly. On multiple occasions, musk deer were also observed curiously watching on camera traps, which may be part of their alert/vigilance behavior.

HMD were primarily observed as solitary individuals throughout the study period, except on rare occasions when adult females were seen with a fawn and during courtship displays involving a male and female. Vocalizations (“e e e” sound) during courtship were documented. In subsequent video footage, similar “e e e” sounds were heard, though musk deer were not observed within the frame. Female musk deer were observed with fawns on multiple occasions. On one occasion, a musk deer nursing the fawn was also recorded. This might be the first record of nursing for *Moschus leucogaster* in the wild (Video S1: Supplementary Photo_video_rare behaviors).

## 5 Discussion

A thorough understanding of the behavior of musk deer is essential for effective management and conservation strategies. However, musk deer behavior remains poorly studied in Nepal, particularly in wild populations. Despite the difficulties of studying the cryptic and elusive musk deer, we have provided a detailed description of Himalayan musk deer behavior in Lapchi Valley, Nepal, using camera trap footage.

HMD are solitary animals and were predominantly recorded alone throughout the study period. However, we did observe multiple events of musk deer in pairs during male–female courtship displays as well as mothers with a fawn. Similar events of female musk deer with a fawn were reported by Singh et al. (2022) in the Annapurna Conservation Area, Nepal, and courtship displays between male and female musk deer in Vietnam (Tran et al. 2025).

HMD were primarily active during sunrise and sunset, indicating a crepuscular activity pattern in Lapchi Valley. Additionally, smaller peaks in activity were observed during nighttime, suggesting nocturnal behavior. This finding aligns with previous studies that have documented the crepuscular and nocturnal behavior of musk deer. In contrast, forest musk deer ( *Moschus berezovskii* ) in captivity have shown activity peaks only at sunrise and sunset (Lin et al. 2023). Furthermore, HMD exhibited negligible activity during midday. In contrast, activity levels were higher during the pre‐rut season, which may indicate increased foraging to accumulate energy reserves before the rutting season and the onset of winter. During rut, the activity was highest during sunrise while it shifted to sunset during the post‐rut period. However, a study conducted at Nepal's Annapurna Conservation Area showed the highest activity occurring at sunset during breeding season (Singh et al. 2022). Likewise, the moderate temporal activity overlap with domestic animals and humans might also explain the HMD's activity shift toward sunset during post‐rut. During post‐rut, the domestic animals showed the highest activity peaks during the morning hours. Domestic animals showed comparatively less activity during sunset, likely since they had moved toward shelter/resting areas as the evening approached. Additionally, HMD showed a bit of activity during nights while almost no night activity is reported for livestock. Such temporal partitioning could represent an avoidance strategy to reduce disturbance or competition for foraging areas. Khadka et al. (2017) reported partition in dietary composition between livestock and HMD.However, this difference might also be attributed to the sample size or other environmental factors.

Recent research has shown that nocturnal activity increases in areas with high predation pressure and human disturbance (Lee et al. 2024). In Lapchi Valley, human activities are prevalent in musk deer habitats, as local communities follow semi‐nomadic lifestyles, and livestock graze in these areas (Koju, Bashyal, and Shah 2021). Additionally, the predation risks are also high in the region. Previous research from the Lapchi valley has documented two apex‐predators: Snow leopard and Common leopard alongside Himalayan wolves sharing the same habitat; added by other carnivores such as red fox and yellow‐throated marten (Koju et al. 2024), increasing the potential risk of predation. Although this study focused on activity patterns, the influence of environmental conditions, anthropogenic disturbances, and interspecific competition was not examined and thus warrants further investigation.

In addition to reproductive periods, environmental drivers such as temperature, snow cover, and precipitation strongly influence ungulate activity budgets and daily movement patterns (Pęksa and Ciach 2018; Cueva‐Hurtado et al. 2024). Although such variables were not quantified in this study due to the absence of site‐specific meteorological data, their effects could interact with reproductive behavior and food availability to shape HMD activity. Future study incorporating climatic covariates and habitat phenology will be critical for disentangling these effects and refining ecological models of musk deer behavior.

HMD spent a significant portion of their time engaged in locomotion, alertness, sniffing, feeding, and excretion. Studies on captive musk deer, however, have reported resting as the most common behavior (Cheng et al. 2008). This difference may be attributed to reduced foraging challenges in captivity compared to the wild. Greater locomotive actions might be attributed to a greater need for foraging and higher vigilance, which would be expected in a natural habitat compared to captivity. Likewise, the higher representation of mobility behaviors might be attributed to potential sampling bias as sampling was focused on active trails.

Male HMD exhibited higher activity budget in locomotion, which suggests that males spend a greater proportion of their time searching for food or patrolling their territories. Previous research has indicated that male musk deer have a larger home range than females (Kattel 1993) and are more territorial, which may explain their increased activity levels. However, no statistically significant difference in the activity budget between male and female HMD was observed.

Various extrinsic and intrinsic factors influence the activity budget of wild animals. In this study, the highest number of behavioral events was recorded during the rutting period. This is partly attributable to sampling design, as camera traps were continuously active throughout the rut season across the sampling years, leading to a higher number of videos captured during this period. Additionally, the physiological and behavioral ecology of musk deer during the rut period, such as heightened competition for territory and searching for mates, naturally increases detection likelihood (Meng et al. 2008). The results from this study showed the activity budget of musk deer varied significantly across different periods of the year (pre‐rut, rut, and post‐rut) in Lapchi Valley. However, we acknowledge that the behavioral observations were not evenly distributed across reproductive periods, with most detections during the rut. This uneven sampling could introduce bias in estimating activity budgets, particularly for post‐rut behaviors. However, a jackknife sensitivity analysis performed on subsample datasets (removing 20% of rut observations iteratively) showed consistent trends in locomotion and vigilance, suggesting robustness of our results. Nonetheless, future study should adopt a stratified temporal sampling design to ensure equal representation across periods.

Furthermore, a significant difference in behavioral events between male and female musk deer was observed during these periods. Male and female musk deer undergo varied physiological mechanisms such as maternal care needs, reproductive effort, or habitat constraints throughout the year, which results in the variation in time spent on different behavioral activities. Previous studies have also reported that individual factors such as age and sex influence behavioral patterns and activity budgets of wild animals over time (Huettner et al. 2021). Female musk deer spent more time on locomotion and less time standing (resting) during the post‐rut period compared to males, likely due to their increased focus on fawn protection and feeding, especially during lactation and possibly due to snowmelt altering forage distribution (Meng et al. 2011).

Male musk deer were found to be more alert/vigilant during the post‐rut period. This coincides with the movement of livestock into musk deer habitats in Lapchi Valley, which increases anthropogenic disturbances. Other anthropogenic pressures, such as road and hydropower construction and tourism, are also growing in the region. These developmental activities likely result in a loss of musk deer habitat. The excessive noise during construction and increased human presence may create a shift to more nocturnal activity in musk deer. Research has shown that external factors such as climatic conditions (Pęksa and Ciach 2018), food availability (Albani et al. 2020), and anthropogenic disturbances such as construction (Jiang et al. 2013; Pęksa and Ciach 2018; Albani et al. 2020; Huettner et al. 2021) can alter the behavioral and activity pattern of wildlife. While there are records of miscellaneous behaviors of musk deer in captivity, this study captured rare and unique behaviors in the wild, providing new insights into their ecology. Behaviors such as self‐grooming, courtship displays, and non‐solitary interactions were documented. Musk deer exhibit olfactory communication through latrine site and tail‐pasting behavior (Singh et al. 2022), however, vocalization is rarely recorded for musk deer. Recently, a profound “e e e” sound was documented by Tran et al. (2025) during mating of the forest musk deer (*Moschus berezovskii*) in the wild. Similarly, this study also documented a similar vocalization (an “e e e” sound) in the HMD, which is possibly associated with courtship vocalizations. Additionally, non‐solitary interactions in musk deer were associated with mating behavior and fawn nurturing. These findings highlight the need for further research on the behavioral ecology of musk deer. This would include a more robust sampling design incorporating a longer study duration and inclusion of GPS‐collaring and acoustic monitoring, thus allowing for a more comprehensive understanding of this rare and elusive species.

## 6 Conclusion

Studying the behavioral activity of musk deer is challenging due to their shy, solitary nature and predominantly crepuscular activity patterns. However, noninvasive sampling techniques such as camera trapping provide valuable opportunities to quantify behavior and time allocation in nature. Using camera trap data, this study provides critical insights into the behavior, activity patterns, and time budget allocation of the Himalayan musk deer (HMD) in the Gaurishankar Conservation Area, Nepal. Behavior of HMD varied significantly across reproductive periods but showed no detectable differences between sexes. The moderate temporal overlap between HMD and domestic livestock suggests behavioral adjustments in response to anthropogenic disturbance, particularly a shift towards evening activity during the post‐rut period. Although the study provided useful data, it was limited by uneven seasonal sampling, limited camera coverage, and inability to record behaviors beyond the cameras' detection range – likely biasing observation behavioral toward more mobile and visible activities. Therefore, future studies should focus broader spatial and temporal coverage, integrate habitat and climate covariates, and extend monitoring across seasonal livestock exclusion periods to provide more comprehensive understanding of behaviroal ecology. Such understanding is essential for developing effective conservation strategies, particularly in the face of increasing anthropogenic pressures. This study contributes to the growing body of knowledge on musk deer behavior and highlights the importance of continued monitoring and research to inform conservation efforts for this endangered species.

## Author Contributions

**Bijay Bashyal:** conceptualization (equal), data curation (equal), formal analysis (equal), funding acquisition (equal), investigation (equal), methodology (equal), resources (equal), software (equal), validation (equal), visualization (equal), writing – original draft (equal), writing – review and editing (equal). **Lila Paudel:** conceptualization (equal), data curation (equal), formal analysis (equal), methodology (equal), software (equal), validation (equal), writing – original draft (equal), writing – review and editing (equal). **Paul Buzzard:** conceptualization (equal), data curation (equal), funding acquisition (equal), investigation (equal), methodology (equal), resources (equal), writing – original draft (equal), writing – review and editing (equal). **Randall C. Kyes:** conceptualization (equal), formal analysis (equal), funding acquisition (equal), methodology (equal), software (equal), supervision (equal), writing – original draft (equal), writing – review and editing (equal). **Narayan Prasad Koju:** conceptualization (equal), data curation (equal), formal analysis (equal), funding acquisition (equal), investigation (equal), methodology (equal), project administration (equal), resources (equal), software (equal), supervision (equal), validation (equal), visualization (equal), writing – original draft (equal), writing – review and editing (equal).

## Funding

This work was supported by the Gaurishankar Conservation Area Project; University Grants Commission‐ Nepal, FRG 77/78 S&T‐01 2020/21, FRG 80/81 S&T 2023/2025; Office of Research Infrastructure Programs (ORIP) of the National Institutes, P51OD010425.

## Conflicts of Interest

The authors declare no conflicts of interest.

## Supporting information


**Data S1:** ece372766‐sup‐0001‐DataS1.rar.
**Table S1:** The number of observations (*n*) and percentage (%) of the total female and male musk deer during the pre‐rut, rut, and post‐rut period in the Lapchi Valley of Gaurishankar Conservation Area, Nepal, Oct 2021–March 2023.
**Table S2:** The sum of durations (minutes) (*t*) and percentage (%) of the total female and male musk deer during the pre‐rut, rut, and post‐rut period in the Lapchi Valley of Gaurishankar Conservation Area, Nepal, Oct 2021–March 2023.
**Table S3:** Chi‐squared tests for variation in frequency of occurrences of behavioral events for female and male musk deer and behaviors category during period (pre‐rut, rut and post‐rut) of year, respectively, in Lapchi valley, Gaurishankar Conservation Area, Nepal.
**Table S4:** Chi‐squared tests for variation in duration (seconds) of behavioral events for female and male musk deer and behaviors category during period (pre‐rut, rut, and post‐rut) of year, respectively, in Lapchi valley, Gaurishankar Conservation Area, Nepal.
**Table S5:** One‐way ANOVA analysis of activity of musk deer (time) observed during the pre‐rut, rut, and post‐rut period, respectively.
**Table S6:** The total number of observations (*n*) and percentage (%) of female and male musk deer during the pre‐rut, rut, and post‐rut period in the Lapchi Valley of Gaurishankar Conservation Area, Nepal, Oct 2021–March 2023.
**Video S1:** Video 11180404: Body shaking (self‐grooming) behavior by male Himalayan Musk Deer Video 11080350: Body shaking (self‐grooming) behavior by female Himalayan Musk Deer Video 11040302: Acoustic signals produced during courtship behavior involving male and female Himalayan Musk Deer Video 11040302: Acoustic signals produced during courtship behavior involving male and female Himalayan Musk Deer Video 12060698: Tail‐pasting behavior by male Himalayan Musk Deer Video 02030039: Response to certain stimuli or threat by male Himalayan Musk Deer Video 06220093: Meeting of two Himalayan Musk Deer individuals Image 06160337; Image 06020195: Female Himalayan Musk Deer along with fawn Image EK000311: A female individual being followed by Male Himalayan Musk Deer Image 08010127: Two individuals of Himalayan Musk Deer in single frame Video 08180144: A Himalayan Musk Deer using fore limbs for digging Video 12010033: Sniffing and Excretion behavior by male Himalayan Musk Deer Video 12030063: Excretion behavior by male Himalayan Musk Deer Image 06010177: A female Himalayan Musk Deer lactating its fawn.

## Data Availability

https://doi.org/10.5281/zenodo.15600012.

## References

[ece372766-bib-0001] Albani, A. , M. Cutini , L. Germani , E. P. Riley , P. O. Ngakan , and M. Carosi . 2020. “Activity Budget, Home Range, and Habitat Use of Moor Macaques (*Macaca maura*) in the Karst Forest of South Sulawesi, Indonesia.” Primates 61, no. 5: 673–684. 10.1007/s10329-020-00811-8. 32170514

[ece372766-bib-0102] Altmann, J. 1974. “Observational Study of Behavior: Sampling Methods.” Behaviour: 227–267. 4597405 10.1163/156853974x00534

[ece372766-bib-0002] Amin, R. , H. S. Baral , B. R. Lamichhane , et al. 2018. “The Status of Nepal's Mammals.” Journal of Threatened Taxa 10, no. 3: 11361–11378. 10.11609/jott.3712.10.3.11361-11378.

[ece372766-bib-0003] Carbone, C. , S. Christie , K. Conforti , et al. 2001. “The Use of Photographic Rates to Estimate Densities of Tigers and Other Cryptic Mammals.” Animal Conservation 4: 81. 10.1017/S1367943001001081.

[ece372766-bib-0004] Cheng, X. , M. Xiuxiang , X. Hongfa , and X. Yu . 2008. “Activity Rhythm and Behavioral Time Budgets of the Captive Forest Musk Deer (*Moschus berezovskii*) in Spring.” Acta Theriologica Sinica 28, no. 2: 194–200. https://www.mammal.cn/EN/Y2008/V28/I2/194.

[ece372766-bib-0005] Chetri, M. , P. R. Regmi , T. P. Dahal , and S. Thami . 2022. “A Checklist of Mammals of Gaurishankar Conservation Area, Nepal.” Nepalese Journal of Zoology 6, no. S1: 56–62. 10.3126/njz.v6iS1.50533.

[ece372766-bib-0006] Christiansen, F. , M. H. Rasmussen , and D. Lusseau . 2013. “Inferring Activity Budgets in Wild Animals to Estimate the Consequences of Disturbances.” Behavioral Ecology 24, no. 6: 1415–1425. 10.1093/beheco/art086.

[ece372766-bib-0007] CITIES . 2023. “Checklist of CITES Species.” https://checklist.cites.org/#/en/search/output_layout=alphabetical&level_of_listing=0&show_synonyms=1&show_author=1&show_english=1&show_spanish=1&show_french=1&scientific_name=elephant&page=1&per_page=20.

[ece372766-bib-0008] Cueva‐Hurtado, L. , A. Jara‐Guerrero , R. Cisneros , and C. I. Espinosa . 2024. “Activity Patterns of the White‐Tailed Deer ( *Odocoileus virginianus* ) in a Neotropical Dry Forest: Changes According to Age, Sex, and Climatic Season.” THERYA 15, no. 2: 242–252. 10.12933/therya-24-5029.

[ece372766-bib-0009] Dhami, B. , N. B. Chhetri , B. Neupane , et al. 2024. “Predicting the Current Habitat Refugia of Himalayan Musk Deer (*Moschus chrysogaster*) Across Nepal.” Ecology and Evolution 14, no. 2: e10949. 10.1002/ece3.10949. 38371859 PMC10870248

[ece372766-bib-0010] Friard, O. , and M. Gamba . 2016. “BORIS: A Free, Versatile Open‐Source Event‐Logging Software for Video/Audio Coding and Live Observations.” Methods in Ecology and Evolution 7, no. 11: 1325–1330. 10.1111/2041-210X.12584.

[ece372766-bib-0011] Ghaskadbi, P. , B. Habib , and Q. Qureshi . 2016. “A Whistle in the Woods: An Ethogram and Activity Budget for the Dhole in Central India.” Journal of Mammalogy 97, no. 6: 1745–1752. 10.1093/jmammal/gyw141.

[ece372766-bib-0012] Ghimire, H. R. , and S. Phuyal . 2022. “Spatiotemporal Analysis of Hydropower Projects With Terrestrial Environmentally Sensitive Areas of Nepal.” Environmental Challenges 9: 100598. 10.1016/j.envc.2022.100598.

[ece372766-bib-0013] Green, M. J. 1986. “The Distribution, Status and Conservation of the Himalayan Musk Deer *Moschus chrysogaster* .” Biological Conservation 35, no. 4: 347–375. 10.1016/0006-3207(86)90094-7.

[ece372766-bib-0014] Green, M. J. 1987. “Scent‐Marking in the Himalayan Musk Deer (*Moschus chrysogaster*).” Journal of Zoology 1, no. 4: 721–737.

[ece372766-bib-0016] Hamel, S. , and S. D. Côté . 2008. “Trade‐Offs in Activity Budget in an Alpine Ungulate: Contrasting Lactating and Nonlactating Females.” Animal Behaviour 75, no. 1: 217–227. 10.1016/j.anbehav.2007.04.028.

[ece372766-bib-0017] Harris, R. B. 2016. “Moschus chrysogaster.” IUCN Red List of Threatened Species 2016: e.T13895A61977139. 10.2305/IUCN.UK.2016.RLTS.T13895A61977139.en.

[ece372766-bib-0018] Howell, C. P. , and S. M. Cheyne . 2019. “Complexities of Using Wild Versus Captive Activity Budget Comparisons for Assessing Captive Primate Welfare.” Journal of Applied Animal Welfare Science 22, no. 1: 78–96. 10.1080/10888705.2018.1500286. 30058408

[ece372766-bib-0019] Huettner, T. , S. Dollhaeupl , R. Simon , K. Baumgartner , and L. von Fersen . 2021. “Activity Budget Comparisons Using Long‐Term Observations of a Group of Bottlenose Dolphins (*Tursiops truncatus*) Under Human Care: Implications for Animal Welfare.” Animals 11, no. 7: 2107. 10.3390/ani11072107. 34359239 PMC8300398

[ece372766-bib-0020] IUCN . 2018. “The IUCN Red List of Threatened Species 2018.” International Union for Conservation of Nature—IUCN. https://www.iucnredlist.org/.

[ece372766-bib-0021] Jiang, T. , X. Wang , Y. Ding , Z. Liu , and Z. Wang . 2013. “Behavioral Responses of Blue Sheep ( *Pseudois nayaur* ) to Nonlethal Human Recreational Disturbance.” Chinese Science Bulletin 58: 2237–2247. 10.1007/s11434-013-5761-y.

[ece372766-bib-0022] Jnawali, S. R. , H. S. Baral , S. Lee , et al. 2011. “The Status of Nepal's Mammals: The National Red List Series.”

[ece372766-bib-0023] Kattel, B. 1993. Ecology of the Himalayan Musk Deer in Sagarmatha National Park. Colorado State University.

[ece372766-bib-0104] Khadka, K. K. , N. Singh , K. T. Magar , and D. A. James . 2017. “Dietary Composition, Breadth, and Overlap Between Seasonally Sympatric Himalayan Musk Deer and Livestock: Conservation Implications.” Journal for Nature Conservation 38: 30–36.

[ece372766-bib-0024] Koju, N. P. , B. Bashyal , B. P. Pandey , S. N. Shah , S. Thami , and W. V. Bleisch . 2021. “First Camera‐Trap Record of the Snow Leopard *Panthera uncia* in Gaurishankar Conservation Area, Nepal.” Oryx 55, no. 2: 173–176. 10.1017/S003060532000006X.

[ece372766-bib-0025] Koju, N. P. , B. Bashyal , B. P. Pandey , S. Thami , M. K. Dhamala , and S. N. Shah . 2020. “New Record on Asiatic Golden Cat *Catopuma temminckii* Vigors & Horsfield, 1827 (Mammalia: Carnivora: Felidae): Photographic Evidence of Its Westernmost Distribution in Gaurishankar Conservation Area, Nepal.” Journal of Threatened Taxa 12, no. 2: 15256–15261. 10.11609/jott.5227.12.2.15256-15261.

[ece372766-bib-0026] Koju, N. P. , B. Bashyal , and S. N. Shah . 2021. “Habitat Preference of Himalayan Musk Deer ( *Moschus leucogaster* Hodgson, 1839) at Lapchi of Bigu Rural Municipality, Gaurishankar Conservation Area.” Nepal Journal of Environmental Science 9, no. 1: 21–28. 10.3126/njes.v9i1.37844.

[ece372766-bib-0027] Koju, N. P. , P. Buzzard , A. Shrestha , et al. 2024. “Habitat Overlap and Interspecific Competition Between Snow Leopards and Leopards in the Central Himalaya of Nepal.” Global Ecology and Conservation 52: e02953. 10.1016/j.gecco.2024.e02953.

[ece372766-bib-0028] Koju, N. P. , K. R. Gosai , B. Bashyal , et al. 2023. “Seasonal Prey Abundance and Food Plasticity of the Vulnerable Snow Leopard (*Panthera uncia*) in the Lapchi Valley, Nepal Himalayas.” Animals 13, no. 20: 3182. 10.3390/ani13203182. 37893906 PMC10603713

[ece372766-bib-0029] Lee, S. X. T. , Z. Amir , J. H. Moore , K. M. Gaynor , and M. S. Luskin . 2024. “Effects of Human Disturbances on Wildlife Behaviour and Consequences for Predator‐Prey Overlap in Southeast Asia.” Nature Communications 15, no. 1: 1521. 10.1038/s41467-024-45905-9. PMC10876642 38374248

[ece372766-bib-0030] Lin, S. , L. Shen , H. Gao , et al. 2023. “The Autumn Activity Patterns and Time Budgets of Forest Musk Deer (*Moschus berezovskii*) in Captivity.” Veterinary Research Forum 14: 589–594. 38169479 10.30466/vrf.2023.1978088.3703 PMC10758006

[ece372766-bib-0031] Lu, L. , P. Yan , X. Meng , et al. 2009. “Behavioral Patterns of Captive Alpine Musk Deer: Sex‐Specific Behavior Comparisons.” Frontiers of Agriculture in China 3: 300–303. 10.1007/s11703-009-0055-5.

[ece372766-bib-0032] Mainali, K. P. , P. B. Singh , M. Evans , A. Adhikari , Y. Hu , and H. Hu . 2023. “A Brighter Shade of Future Climate on Himalayan Musk Deer *Moschus leucogaster* .” Scientific Reports 13, no. 1: 12771. 10.1038/s41598-023-39481-z. 37550330 PMC10406878

[ece372766-bib-0033] Meng, X. , Q. Yang , Z. Feng , et al. 2008. “Seasonal Behavioral Patterns of Captive Alpine Musk Deer (*Moschus sifanicus*): Rut and Pre‐Rut Comparisons.” Biologia 63: 594–598. 10.2478/s11756-008-0085-0.

[ece372766-bib-0034] Meng, X. , C. Zhao , C. Hui , and X. Luan . 2011. “Behavioral Aspects of Captive Alpine Musk Deer During Non‐Mating Season: Gender Differences and Monthly Patterns.” Asian‐Australasian Journal of Animal Sciences 24, no. 5: 707–712. 10.5713/ajas.2011.10425.

[ece372766-bib-0035] Meredith, M. , and M. Ridout . 2014. “overlap: Estimates of Coefficient of Overlapping for Animal Activity Patterns.” R Package Version 0.2, 4. 10.1198/jabes.2009.08038.

[ece372766-bib-0036] Miller, L. J. , G. A. Vicino , J. Sheftel , and L. K. Lauderdale . 2020. “Behavioral Diversity as a Potential Indicator of Positive Animal Welfare.” Animals 10, no. 7: 1211. 10.3390/ani10071211. 32708625 PMC7401597

[ece372766-bib-0037] Noor, A. , Z. R. Mir , G. G. Veeraswami , and B. Habib . 2017. “Activity Patterns and Spatial Co‐Occurrence of Sympatric Mammals in the Moist Temperate Forest of the Kashmir Himalaya, India.” Folia Zoologica 66, no. 4: 231–241. 10.25225/fozo.v66.i4.a4.2017.

[ece372766-bib-0038] NTNC . 2013. “Protected Areas and Ecosystems.” The National Trust for Nature Conservation (NTNC). https://ntnc.org.np/project/gaurishankar‐conservation‐area‐project‐gcap.

[ece372766-bib-0039] Pęksa, Ł. , and M. Ciach . 2018. “Daytime Activity Budget of an Alpine Ungulate (Tatra Chamois *Rupicapra rupicapra tatrica* ): Influence of Herd Size, Sex, Weather and Human Disturbance.” Mammal Research 63, no. 4: 443–453. 10.1007/s13364-018-0376-y.

[ece372766-bib-0040] R Core Team . 2022. “RStudio (Version 4.2. 1.).”

[ece372766-bib-0041] Ridout, M. S. , and M. Linkie . 2009. “Estimating Overlap of Daily Activity Patterns From Camera Trap Data.” Journal of Agricultural, Biological, and Environmental Statistics 14, no. 3: 322–337. 10.1198/jabes.2009.08038.

[ece372766-bib-0042] Sathyakumar, S. , S. Gopal , and A. Johnsingh . 1992. “The Musk Deer.” Sanctuary Asia 12, no. 5: 52–57.

[ece372766-bib-0103] Seyrling, I. , P. W. Dierkes , and A. L. Burger . 2022. “Diurnal and Nocturnal Behaviour of Cheetahs (*Acinonyx jubatus*) and Lions (*Panthera leo*) in Zoos.” Animals 12, no. 18: 2367. 36139229 10.3390/ani12182367 PMC9495184

[ece372766-bib-0101] Shrestha, B. , J. Khatiwada , and D. Thanet . 2018. “mtDNA Confirms the Presence of *Moschus leucogaster* (Ruminantia, Moschidae) in Gaurishankar Conservation Area, Nepal.” Miscel·Lània Zoològica 17: 209–218.

[ece372766-bib-0043] Singh, P. B. , K. Mainali , Z. Jiang , et al. 2020. “Projected Distribution and Climate Refugia of Endangered Kashmir Musk Deer *Moschus cupreus* in Greater Himalaya, South Asia.” Scientific Reports 10, no. 1: 1511. 32001721 10.1038/s41598-020-58111-6 PMC6992763

[ece372766-bib-0044] Singh, P. B. , P. Saud , Z. Jiang , Z. Zhou , Y. Hu , and H. Hu . 2022. “Himalayan Musk Deer (*Moshcus leucogaster*) Behavior at Latrine Sites and Their Implications in Conservation.” Ecology and Evolution 12, no. 4: e8772. 10.1002/ece3.8772. 35432920 PMC9001115

[ece372766-bib-0045] Subedi, A. , A. Aryal , R. K. Koirala , Y. P. Timilsina , X. Meng , and F. McKenzie . 2012. “Habitat Ecology of Himalayan Musk Deer (*Moschus chrysogaster*) in Manaslu Conservation Area, Nepal.” International Journal of Zoological Research 8, no. 2: 81.

[ece372766-bib-0046] Sutherland, W. J. 1998. “The Importance of Behavioural Studies in Conservation Biology.” Animal Behaviour 56, no. 4: 801–809. 10.1006/anbe.1998.0896. 9790690

[ece372766-bib-0047] Timmins, R. J. , and J. W. Duckworth . 2015a. “*Moschus cupreus*.” The IUCN Red List of Threatened Species 2015: e.T136750A61979453. 10.2305/IUCN.UK.2015-2.RLTS.T136750A61979453.en.

[ece372766-bib-0048] Timmins, R. J. , and J. W. Duckworth . 2015b. “*Moschus leucogaster*.” The IUCN Red List of Threatened Species 2015. 10.2305/IUCN.UK.2015-4.RLTS.T13896A61977357.en.

[ece372766-bib-0049] Tiwari, A. 2022. “Chasing Performance of Protected Area Management in Nepal.” Journal of Tourism and Himalayan Adventures 4, no. 1: 1–16.

[ece372766-bib-0051] Tran, D. V. , D. V. Phan , T. T. Vu , et al. 2025. “First Observation of Mating Behavior of the Endangered Forest Musk Deer *Moschus berezovskii* in the Wild.” Tropical Zoology 37: 186. 10.4081/tz.2024.186.

[ece372766-bib-0052] Wang, Y. , and R. Harris . 2015. “*Moschus fuscus.”* The IUCN Red List of Threatened Species 2015. 10.2305/IUCN.UK.2015-4.RLTS.T13896A61977357.en.

[ece372766-bib-0053] Worku, E. A. , A. Atickem , J. Bro‐Jørgensen , A. Bekele , P. Evangelista , and N. C. Stenseth . 2021. “Human Activities Increase Vigilance, Movement and Home Range Size of the Endangered Mountain Nyala ( *Tragelaphus buxtoni* ) at the Cost of Foraging and Resting.” Global Ecology and Conservation 32: e01900. 10.1016/j.gecco.2021.e01900.

[ece372766-bib-0054] Yang, Q. , X. Meng , L. Xia , and Z. Feng . 2003. “Conservation Status and Causes of Decline of Musk Deer (*Moschus* spp.) in China.” Biological Conservation 109, no. 3: 333–342. 10.1016/S0006-3207(02)00159-3.

