# Supplementary material for: Activity Budget and Behavioral Patterns of Himalayan Musk Deer in Gaurishankar Conservation Area, Nepal
Source: Ecol Evol. 2026 Jan 5;16(1):e72766. doi: 10.1002/ece3.72766 (PMC12771587; doi:10.1002/ece3.72766)
Supplement: Supplementary file 1 — Data S1: ece372766‐sup‐0001‐DataS1.rar. Table S1: The number of observations (n) and percentage (%) of the total female and male musk deer during the pre‐rut, rut, and post‐rut period in the Lapchi Valley of Gaurishankar Conservation Area, Nepal, Oct 2021–March 2023. Table S2: The sum of durations (minutes) (t) and percentage (%) of the total female and male musk deer during the pre‐rut, rut, and post‐rut period in the Lapchi Valley of Gaurishankar Conservation Area, Nepal, Oct 2021–March 2023. Table S3: Chi‐squared tests for variation in frequency of occurrences of behavioral events for female and male musk deer and behaviors category during period (pre‐rut, rut and post‐rut) of year, respectively, in Lapchi valley, Gaurishankar Conservation Area, Nepal. Table S4: Chi‐squared tests for variation in duration (seconds) of behavioral events for female and male musk deer and behaviors category during period (pre‐rut, rut, and post‐rut) of year, respectively, in Lapchi valley, Gaurishankar Conservation Area, Nepal. Table S5: One‐way ANOVA analysis of activity of musk deer (time) observed during the pre‐rut, rut, and post‐rut period, respectively. Table S6: The total number of observations (n) and percentage (%) of female and male musk deer during the pre‐rut, rut, and post‐rut period in the Lapchi Valley of Gaurishankar Conservation Area, Nepal, Oct 2021–March 2023. Video S1: Video 11180404: Body shaking (self‐grooming) behavior by male Himalayan Musk Deer Video 11080350: Body shaking (self‐grooming) behavior by female Himalayan Musk Deer Video 11040302: Acoustic signals produced during courtship behavior involving male and female Himalayan Musk Deer Video 11040302: Acoustic signals produced during courtship behavior involving male and female Himalayan Musk Deer Video 12060698: Tail‐pasting behavior by male Himalayan Musk Deer Video 02030039: Response to certain stimuli or threat by male Himalayan Musk Deer Video 06220093: Meeting of two Himalayan Musk Deer individuals [file ECE3-16-e72766-s001.zip › ece372766-sup-0003-Supinfo03.docx]

**Table S1:** The number of observations (n) and percentage (%) of the total female and male musk deer during the pre-rut, rut and post rut period in the Lapchi Valley of Gaurishankar Conservation Area, Nepal, Oct 2021 – March 2023

|  | **Pre-Rut (Aug, Sept, Oct)** | | | | | | **Rut (Nov, Dec, Jan)** | | | | | | | | **Post Rut (Feb, March, April)** | | | | | |
| --- | --- | --- | --- | --- | --- | --- | --- | --- | --- | --- | --- | --- | --- | --- | --- | --- | --- | --- | --- | --- |
|  | **Female** | | **Male** | | **Total** | | **Female** | | **Male** | | | **Total** | | **Female** | | | **Male** | | **Total** | |
| **Behaviors** | **n** | **%** | **n** | **%** | **n** | **%** | **n** | **%** | **n** | **%** | **n** | | **%** | **n** | | **%** | **n** | **%** | **n** | **%** |
| locomotion | 30 | 19.6 | 14 | 9.2 | 44 | 28.8 | 77 | 12.6 | 107 | 17.6 | 184 | | 30.2 | 11 | | 9.0 | 29 | 23.8 | 40 | 32.8 |
| alert/vigilance | 11 | 7.2 | 2 | 1.3 | 13 | 8.5 | 32 | 5.3 | 35 | 5.7 | 67 | | 11.0 | 6 | | 4.9 | 8 | 6.6 | 14 | 11.5 |
| excrete | 2 | 1.3 | 2 | 1.3 | 4 | 2.6 | 4 | 0.7 | 10 | 1.6 | 14 | | 2.3 | 1 | | 0.8 | 1 | 0.8 | 2 | 1.6 |
| feed | 14 | 9.2 | 4 | 2.6 | 18 | 11.8 | 25 | 4.1 | 21 | 3.4 | 46 | | 7.6 | 2 | | 1.6 | 14 | 11.5 | 16 | 13.1 |
| out of sight | 10 | 6.5 | 5 | 3.3 | 15 | 9.8 | 26 | 4.3 | 38 | 6.2 | 64 | | 10.5 | 4 | | 3.3 | 11 | 9.0 | 15 | 12.3 |
| ruminate | 1 | 0.7 | 0 | 0.0 | 1 | 0.7 | 5 | 0.8 | 6 | 1.0 | 11 | | 1.8 | 0 | | 0.0 | 2 | 1.6 | 2 | 1.6 |
| sniff | 14 | 9.2 | 4 | 2.6 | 18 | 11.8 | 28 | 4.6 | 33 | 5.4 | 61 | | 10.0 | 2 | | 1.6 | 9 | 7.4 | 11 | 9.0 |
| stand | 26 | 17.0 | 10 | 6.5 | 36 | 23.5 | 68 | 11.2 | 81 | 13.3 | 149 | | 24.5 | 6 | | 4.9 | 16 | 13.1 | 22 | 18.0 |
| Miscellaneous | 4 | 2.6 | 0 | 0.0 | 4 | 2.6 | 6 | 1.0 | 7 | 1.1 | 13 | | 2.1 | 0 | | 0.0 | 0 | 0.0 | 0 | 0.0 |
| Grand Total | 112 | 73.2 | 41 | 26.8 | 153 | 100.0 | 271 | 44.5 | 338 | 55.5 | 609 | | 100.0 | 32 | | 26.2 | 90 | 73.8 | 122 | 100.0 |

**Table S2:** The sum of durations (minutes) (t) and percentage (%) of the total female and male musk deer during the pre-rut, rut and post rut period in the Lapchi Valley of Gaurishankar Conservation Area, Nepal, Oct 2021 – March 2023

|  | **Pre-Rut (Aug, Sept, Oct)** | | | | | | | **Rut (Nov, Dec,Jan)** | | | | | | | **Post Rut (Feb, March, April)** | | | | | | |  |
| --- | --- | --- | --- | --- | --- | --- | --- | --- | --- | --- | --- | --- | --- | --- | --- | --- | --- | --- | --- | --- | --- | --- |
|  | **Female** | | **Male** | | **Total** | | | **Female** | | **Male** | | **Total** | | | **Female** | | **Male** | | | **Total** | |  |
| **Behaviors** | **n** | **%** | **n** | **%** | | **n** | **%** | **n** | **%** | **n** | **%** | | **n** | **%** | **n** | **%** | | **n** | **%** | **n** | **%** | |
| locomotion | 1.4 | 19.2 | 0.6 | 8.7 | | 2.0 | 27.9 | 5.1 | 11.2 | 8.1 | 17.8 | | 13.3 | 29.0 | 0.9 | 8.6 | | 3.4 | 31.7 | 4.3 | 40.4 | |
| alert/vigilance | 0.4 | 5.3 | 0.1 | 0.8 | | 0.4 | 6.1 | 1.6 | 3.5 | 2.2 | 4.8 | | 3.8 | 8.2 | 0.1 | 1.1 | | 0.9 | 8.5 | 1.0 | 9.6 | |
| excrete | 0.1 | 1.4 | 0.0 | 0.5 | | 0.1 | 1.8 | 0.2 | 0.5 | 1.7 | 3.7 | | 1.9 | 4.2 | 0.0 | 0.1 | | 0.2 | 1.6 | 0.2 | 1.7 | |
| feed | 0.4 | 5.7 | 0.1 | 1.6 | | 0.5 | 7.2 | 0.9 | 1.9 | 1.2 | 2.5 | | 2.0 | 4.5 | 0.1 | 1.0 | | 0.7 | 6.6 | 0.8 | 7.7 | |
| miscellaneous | 0.2 | 2.1 | 0.0 | 0.0 | | 0.2 | 2.1 | 0.1 | 0.3 | 0.4 | 0.8 | | 0.5 | 1.1 | 0.0 | 0.0 | | 0.0 | 0.0 | 0.0 | 0.0 | |
| out of sight | 0.7 | 9.3 | 0.4 | 5.6 | | 1.1 | 14.9 | 2.7 | 5.9 | 3.7 | 8.1 | | 6.4 | 14.0 | 0.9 | 8.3 | | 1.1 | 10.7 | 2.0 | 19.0 | |
| ruminate | 0.1 | 1.1 | 0.0 | 0.0 | | 0.1 | 1.1 | 0.3 | 0.8 | 0.4 | 0.8 | | 0.7 | 1.6 | 0.0 | 0.0 | | 0.1 | 1.2 | 0.1 | 1.2 | |
| sniff | 0.5 | 6.4 | 0.3 | 3.8 | | 0.7 | 10.2 | 1.4 | 3.0 | 1.6 | 3.4 | | 2.9 | 6.4 | 0.0 | 0.4 | | 0.3 | 2.7 | 0.3 | 3.1 | |
| stand | 1.6 | 22.4 | 0.4 | 6.2 | | 2.1 | 28.6 | 7.2 | 15.8 | 7.0 | 15.2 | | 14.2 | 31.0 | 0.2 | 1.8 | | 1.6 | 15.5 | 1.8 | 17.3 | |
| total (sec) | 5.3 | 72.9 | 2.0 | 27.1 | | 7.2 | 100.0 | 19.6 | 42.8 | 26.1 | 57.2 | | 45.7 | 100.0 | 2.3 | 21.3 | | 8.3 | 78.7 | 10.6 | 100.0 | |

**Table S3:** Chi-Squared Tests for variation in frequency of occurrences of behavioral events for female and male musk deer and behaviors category during period (pre-rut, rut and post-rut) of year respectively in Lapchi valley, Gaurishankar Conservation Area, Nepal.

1. Between sex of musk deer and period of year

| **Subject** | **period** | | | **Total** |  | **Chi-Squared Tests** | | | |
| --- | --- | --- | --- | --- | --- | --- | --- | --- | --- |
|  | **post rut** | **pre rut** | **rut** |  |  |  | **Value** | **df** | **p** |
| Female | 32 | 112 | 271 | 415 |  | Χ² | 64.836 | 2 | < .001 |
| Male | 90 | 41 | 338 | 469 |  | N | 884 |  |  |
| Total | 122 | 153 | 609 | 884 |  |  |  |  |  |

1. Between behavior category and period of year

| **Behavior** | **period** | | | **Total** |  | **Chi-Squared Tests** | | | |
| --- | --- | --- | --- | --- | --- | --- | --- | --- | --- |
|  | **post rut** | **pre rut** | **rut** |  |  |  | **Value** | **df** | **p** |
| Locomotion | 40 | 44 | 184 | 268 |  | Χ² | 13.138 | 16 | 0.663 |
| Miscellaneous | 0 | 4 | 13 | 17 |  | N | 884 |  |  |
| alert/vigilance | 14 | 13 | 67 | 94 |  |  |  |  |  |
| excrete | 2 | 4 | 14 | 20 |  |  |  |  |  |
| feed | 16 | 18 | 46 | 80 |  |  |  |  |  |
| out of sight | 15 | 15 | 64 | 94 |  |  |  |  |  |
| ruminate | 2 | 1 | 11 | 14 |  |  |  |  |  |
| sniff | 11 | 18 | 61 | 90 |  |  |  |  |  |
| stand | 22 | 36 | 149 | 207 |  |  |  |  |  |
| Total | 122 | 153 | 609 | 884 |  |  |  |  |  |

**Table S4:** Chi-Squared Tests for variation in duration (seconds) of behavioral events for female and male musk deer and behaviors category during period (pre-rut, rut and post-rut) of year respectively in Lapchi valley, Gaurishankar Conservation Area, Nepal.

1. Between sex of musk deer and period of year

| **Subject** | **period** | | | **Total** |  | **Chi-Squared Tests** | | | |
| --- | --- | --- | --- | --- | --- | --- | --- | --- | --- |
|  | **post rut** | **pre rut** | **rut** |  |  |  | **Value** | **df** | **p** |
| Female | 135.336 | 316.426 | 1173.675 | 1625.437 |  | Χ² | 281.061 | 2 | < .001 |
| Male | 500.544 | 117.651 | 1566.818 | 2185.013 |  | N | 3810.45 |  |  |
| Total | 635.88 | 434.077 | 2740.493 | 3810.45 |  |  | | | |

1. Between behavior category and period of year

| **Behavior** | **period** | | | **Total** |  | **Chi-Squared Tests** | | | |
| --- | --- | --- | --- | --- | --- | --- | --- | --- | --- |
|  | **post rut** | **pre rut** | **rut** |  |  |  | **Value** | **df** | **p** |
| Locomotion | 256.584 | 121.284 | 795.585 | 1173.453 |  | Χ² | 128.855 | 16 | < .001 |
| Miscellaneous | 0 | 9.065 | 29.745 | 38.81 |  | N | 3810.45 |  |  |
| alert/vigilance | 61.037 | 26.461 | 225.616 | 313.114 |  |  | | | |
| excrete | 10.865 | 7.966 | 115.738 | 134.569 |  |  |  |  |  |
| feed | 48.67 | 31.447 | 122.539 | 202.656 |  |  |  |  |  |
| out of sight | 120.786 | 64.511 | 383.549 | 568.846 |  |  |  |  |  |
| ruminate | 7.773 | 4.734 | 42.86 | 55.367 |  |  |  |  |  |
| sniff | 19.999 | 44.343 | 174.549 | 238.891 |  |  |  |  |  |
| stand | 110.166 | 124.266 | 850.312 | 1084.744 |  |  |  |  |  |
| Total | 635.88 | 434.077 | 2740.493 | 3810.45 |  |  |  |  |  |

**Table S5:** One-way ANOVA analysis of activity of musk deer (time) observed during the pre-rut, rut, and post-rut period respectively

| **ANOVA - time** |  |  |  |  |  |
| --- | --- | --- | --- | --- | --- |
| **Cases** | **Sum of Squares** | **df** | **Mean Square** | **F** | **p** |
| period | 0.277 | 2 | 0.138 | 1.386 | 0.251 |
| Residuals | 54.817 | 549 | 0.1 |  |  |
| *Note: Type III Sum of Squares* |  |  |  |  |  |
|  |  |  |  |  |  |

Table S6: The total number of observations (n) and percentage (%) of female and male musk deer during the pre-rut, rut and post rut period in the Lapchi Valley of Gaurishankar Conservation Area, Nepal, Oct 2021 – March 2023

|  | **Pre-Rut period** | | | | | | **Rut period** | | | | | | **Post Rut period** | | | | | |
| --- | --- | --- | --- | --- | --- | --- | --- | --- | --- | --- | --- | --- | --- | --- | --- | --- | --- | --- |
|  | **Female** | | **Male** | | **Total** | | **Female** | | **Male** | | **Total** | | **Female** | | **Male** | | **Total** | |
| **Behaviors** | **n** | **%** | **n** | **%** | **n** | **%** | **n** | **%** | **n** | **%** | **n** | **%** | **n** | **%** | **n** | **%** | **n** | **%** |
| Locomotion | 30 | 19.6 | 14 | 9.2 | 44 | 28.8 | 77 | 12.6 | 107 | 17.6 | 184 | 30.2 | 11 | 9.0 | 29 | 23.8 | 40 | 32.8 |
| Alert/vigilance | 11 | 7.2 | 2 | 1.3 | 13 | 8.5 | 32 | 5.3 | 35 | 5.7 | 67 | 11.0 | 6 | 4.9 | 8 | 6.6 | 14 | 11.5 |
| Excrete | 2 | 1.3 | 2 | 1.3 | 4 | 2.6 | 4 | 0.7 | 10 | 1.6 | 14 | 2.3 | 1 | 0.8 | 1 | 0.8 | 2 | 1.6 |
| Feed | 14 | 9.2 | 4 | 2.6 | 18 | 11.8 | 25 | 4.1 | 21 | 3.4 | 46 | 7.6 | 2 | 1.6 | 14 | 11.5 | 16 | 13.1 |
| Out of sight | 10 | 6.5 | 5 | 3.3 | 15 | 9.8 | 26 | 4.3 | 38 | 6.2 | 64 | 10.5 | 4 | 3.3 | 11 | 9.0 | 15 | 12.3 |
| Ruminate | 1 | 0.7 | 0 | 0.0 | 1 | 0.7 | 5 | 0.8 | 6 | 1.0 | 11 | 1.8 | 0 | 0.0 | 2 | 1.6 | 2 | 1.6 |
| Sniff | 14 | 9.2 | 4 | 2.6 | 18 | 11.8 | 28 | 4.6 | 33 | 5.4 | 61 | 10.0 | 2 | 1.6 | 9 | 7.4 | 11 | 9.0 |
| Stand | 26 | 17.0 | 10 | 6.5 | 36 | 23.5 | 68 | 11.2 | 81 | 13.3 | 149 | 24.5 | 6 | 4.9 | 16 | 13.1 | 22 | 18.0 |
| Miscellaneous | 4 | 2.6 | 0 | 0.0 | 4 | 2.6 | 6 | 1.0 | 7 | 1.1 | 13 | 2.1 | 0 | 0.0 | 0 | 0.0 | 0 | 0.0 |
| Grand total | 112 | 73.2 | 41 | 26.8 | 153 | **100** | 271 | 44.5 | 338 | 55.5 | 609 | **100** | 32 | 26.2 | 90 | 73.8 | 122 | **100** |
